# Sudden asystole during radiofrequency ablation: a case report and literature review

**DOI:** 10.1186/1756-0500-7-351

**Published:** 2014-06-10

**Authors:** He-Sheng Hu, Mei Xue, Rui Xu, Xiao-jun Wang, Ming-you Chen, Su-Hua Yan

**Affiliations:** 1 Department of Cardiology, Shandong Provincial Qianfoshan Hospital, Shandong University, 250014 Jinan, PR China

**Keywords:** Radiofrequency ablation, Asystole, Vagal response, Ganglionated plexus

## Abstract

**Background:**

Radiofrequency (RF) ablation is a widely accepted and ideal therapeutic tool to cure some tachycardias. The occurrence of complications varies depending on the procedure being performed. Sudden unexpected prolonged asystole is rare for most ablation procedures and the underlying mechanisms remain unclear.

**Case presentation:**

A case of sudden prolonged asystole induced by RF ablation of a concealed left free wall accessory in a 59-year-old woman with recurrent tachycardia. RF application provoked progressive slowing of the sinus rhythm and then a 13.2-second period of asystole ensued. Asystole was self-healing and no complications were seen in the following follow-up.

**Conclusions:**

RF ablation may develop prolonged asystole due to vagus response caused by stimulation of unmyelinated vagal C-fibers or ganglionated plexus (GP). Reflexible asystole is reproducible and resolves independently, without affecting the procedure of RF ablation.

## Background

Radiofrequency (RF) ablation has revolutionized treatment of arrhythmias and is now considered to be a first-line therapy for some tachycardias [1]. RF is curative, long-term medication is not required. Its occurrence of complications varies depending on the procedure being performed, and serious complications are rare for most ablation procedure [2]. Bradycardia with hypotension is common during ablation procedures, chiefly due to cardiac reflexes stimulated by chemoreceptors or mechanoreceptors found throughout the vessels, atrium, ventricle, pericardium, and coronary arteries. This cardio-inhibitory reflex can increase parasympathetic tone and cause bradycardia, hypotension, and even cardiac asystole. To the best of our knowledge, sudden unexpected asystole has been rare in the past three decades, spanning the time since the first report of successful RF ablation of tachyarrhythmia. However, the actual incidence of asystole due to RF is unknown.

Here, we present a case of prolonged asystolic episode induced by RF ablation of a concealed left free wall accessory pathway through a transaortic approach. We also briefly review previously reported cases to explore the mechanism of sudden asystole and its clinical significance.

## Case presentation

**Figure 1:**
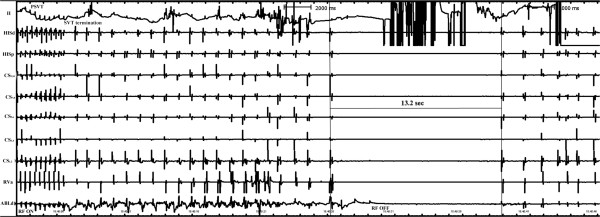
ECG (lead II) and intracardiac electrogram showed an asystole of 13.2 s during radiofrequency energy applications at the atrial aspect of the mitral annulus for the left anterolateral accessory pathway.

A 59-year-old female patient presented with complaints of recurrent palpitations over the past three years. Paroxysmal supraventricular tachycardia (SVT) was diagnosed on several emergency department visits. A 12-lead surface electrogram and physical examination were normal. The SVT had a narrow QRS-complex during the attacks (230 bpm). After informed consent was obtained, the patient received an electrophysiologic study (EPS) and catheter ablation. Multipolar electrode catheters (Biosense Webster, USA) were positioned in the His-bundle region, right ventricular apex, and coronary sinus (CS). Prior to EPS, blood pressure was 121/67 mm Hg and pulse was 72 bpm. Programmed atrial stimulation indicated that sinus and atrioventricular (AV) nodal functions were normal. CS_1,2_ proceeded during ventricular extrastimulus testing and a basic drive (S_1_S_1_) cycle length of 400 ms of ventricular pacing induced AV re-entrant tachycardia (AVRT). The ventriculoatrial (VA) conduction during ventricular pacing was consistent with inducible AVRT. A concealed left free wall accessory pathway was confirmed and selected for mapping and ablation via a transaortic approach. The target site was obtained above the mitral annulus during AVRT and RF energy was delivered (60°C; 30 W) using a temperature-controlled catheter (Biosense Webster). Tachycardia terminated after a 2-sec ablation and ablation was continued for 25 sec more. At 22 sec after the onset of energy delivery, sudden cardiac asystole lasted for 13.2 sec followed by profound sinus rate slowing though ablation was aborted 2 sec after asystole emerged (Figure 1). No escape rhythm emerged. Moreover, AV interval was not changed before and after asystole. After sinus rhythm was rescued, blood pressure was 165/97 mm Hg and ventricular pacing indicated VA disassociation. Multiple ventricular pacing and programmed electrical stimuli for a subsequent 30 min revealed VA disassociation and no induction of tachycardia. Sinus node function AV conduction was normal. The patient no longer experienced tachycardia and syncope 9 months post-ablation.

### Discussion

Here, we describe a rare complication during RF ablation above the mitral annulus during a retrograde aortic approach to ablate a left free wall accessory pathway, characterized by prolonged asystole which resolved on its own. Prolonged asystole has been reported to occur not only occurred during SVT ablation [3-7], but atrial fibrillation ablation [8-10].

### Clinical characteristics

Based on prior case reports and this case, prolonged asystole induced by ablation can be described as depicted in Table 1. These have occurred in individuals with no apparent structural heart disease. Prolonged asystole can occur during slow pathway ablation, at the pulmonary vein, the intra-coronary sinus and around the mitral annulus. Asystole is prone to be reproducible under ablation procedures and Table 1 depicts the duration of each asystolic event (mean = 9.6 ± 3.9 sec) as well as other symptoms patients experienced seemed to resolve on its own and not affect success of ablation. Asystole that does not influence the ablation procedure but may require pacing or atropine. Among the current 10 cases, only one patient failed because the patient requested to complete the process due to anxiety after the second asystole [7].

**Table 1:** Features of 10 Cases of Prolonged Asystole induced by radiofrequency ablation

**Ref. number**	**Age(yrs)/sex**	**Diagnosis**	**Ablation site**	**Asystole time(s)**	**Repeated asystole**	**Therapy**	**Accompanying syndrome**	**Result**	**Adjacent ganglionated plexus**
[8]	45/F	PAF	LSPV	8.2	No mention	Ventricular pacing	No mention	S	LSAGP
[8]	80/M	PAF	LSPV	9.5	No mention	Ventricular pacing	No mention	S	LSAGP
[9]	54/F	PAF	LSPV	17	No mention	None	Syncope	S	LSAGP
[3]	35/M	AVRT	Coronary sinus	8	Y	None	Near fainting; slight pain	S	PLLAGP
[4]	28/F	AVRT	LVFW	5.5	Y	Ventricular pacing	Near syncope; mild pain	S	PLLAGP
[5]	69/F	AVNRT	SP	10.3	Y	Atropine	No discomfort	S	PMLAGP
[6]	52/F	AVNRT	SP	4.6	Y	None	No pain	S	PMLAGP
[7]	43/M	AVNRT	SP	6.9	Y	None	Mild chest pain	F	PMLAGP
[10]	67/M	PAF	LSPV	12.5	Y	Ventricular pacing	No mention	S	LSAGP
Present	59/F	AVRT	LVFW	13.2	N	None	Syncope	S	PLLAGP

*Abbreviations*: *AVNRT* atrioventricular nodal reentrant tachycardia, *AVRT* atrioventricular reentrant tachycardia, *PAF* paroxysmal atrial fibrillation, *SP* slow pathway, *LVFW* left ventricular free wall, *LSPV* left superior pulmonary, *Y* Yes, *N* No, *S* Success, *F* Failure, *LSAGP* Left superior atrial ganglionated plexus, *PMLAGP* posteromedial left atrial ganglionated plexus, *PLLAGP* posterolateral left atrial ganglionated plexus.

### Mechanism

Cardiac asystole has been observed during ablation of various arrhythmias and some intra-cardiac procedures and no clear mechanistic explanation for these events is available. A Bezold–Jarisch-like reflex was a putative mechanism because this is thought to be a cardiovascular decompressor reflex originating in sensory receptors with vagal efferent discharge to the heart [11]. RF current delivery may acutely stimulate the unmyelinated vagal C-fibers which are predominantly distributed within the right atrium lateral wall, the left atrium roof, around the four pulmonary veins, and in the posterior left ventricle. These fibers reflexively increase parasympathetic tone, decrease sympathetic activity, mediate bradycardia-hypotension, and then cause prolonged asystole.

However, bradycardia only lasts 1 to 2 sec after cessation of direct efferent vagal fiber stimulation [12] which is unlike prolonged asystole which persisted for up to 3 sec after cessation of RF current in the series case. Another potential mechanism is the ganglionated plexus (GP) response. GPs embed within epicardial fat pads and the ligament of Marshall, receiving input from the extrinsic cardiac nervous system. This response includes afferent neurons, post-ganglionic efferent parasympathetic and sympathetic neurons, and numerous interconnecting neurons that provide communication within and between the GPs [13]. Capulzini and co-workers reported that targeting the area corresponding to the left superior GP provoked induction of atrial fibrillation and a 12.5-sec asystole [9]. Mathuria and his colleagues speculated that attempted slow-pathway ablation might stimulate nearby GPs and induce cardiac asystole [6]. Whether the GP response is a plausible mechanism that causes reflexive cardio-inhibition is uncertain, evidences suggest that prolonged asystole can be induced by direct GP stimulation. First, targeted GP high-frequency stimulation (HFS) produced a notable vagal response including prolonged asystole. GP ablation has been found to be effective in the treatment of AF and HFS is commonly used to verify localization and ablation of GP via evoked vagal reflexes [14]. HFS of GPs elicited 3.1 sec ventricular asystole via an endocardial approach [15] and a 7.85 sec AV block in the left atrial epicardium prior to RF ablation in patients with AF [16]. Secondly, GPs are composed of sympathetic and parasympathetic nerves, the majority of which are cholinergic [17]. Stimulation of GPs predominantly elicited more vagal effects, as seen in targeted GP ablation. Thirdly, ablation sites are close to the GP area (Table 1). Slow pathway ablation most likely corresponded with the interatrial septal GP formed by the two posterior atrial GPs that are fused and extend anteriorly into the interatrial septum. Ablation of the coronary sinus and left superior pulmonary (LSPV) occurs in areas adjacent to the posterolateral left atrial GP and the superior left atrial GP respectively. Moreover, asystole may occur more commonly in the left atrium and the interatrial septum, which has abundant GPs (60%, 3/5). Tsai et al. [8] reported that 15% (6/40) of patients developed bradycardia-hypotension syndrome during energy delivery to the superior pulmonary veins for treatment of paroxysmal atrial fibrillation. The present case may be caused by stimulation of GP or its adjacent area which facilitated sudden cardiac asystole during attempted ablation in the region of left free wall accessory pathway. Moreover, pain and other factors (nausea, sedation and anxiety) may also be associated with the response. In these collected cases, 3 patients (3/10, 30%) showed chest pain. Clinically, RF induced pain was common and slight. And asystole still emerged repeatedly though the patient was familiar with the procedure and pain [3]. The causal mechanism is sometimes implicated in clinical situations and study should be furthered to disclose the intrinsic mechanism.

## Conclusions

However, profound sinus rate slowing and no AV interval changing during asystole may imply preferential sinus node effects. The intrinsic cardiac nervous system is very complex and its precise anatomy has not been explored in detail. The above mentioned causal mechanisms can only be speculative. The exact nature involved is not unclear. RF ablation may develop prolonged asystole due to vagus response caused by stimulation of unmyelinated vagal C-fibers or GP. Although prior case reports have shown the reflexible asystole may not affect RF ablation procedures, clinical cardiologists should be aware of this rare complication during RF ablation and properly manage these events, especially ablating the GP and its adjacent area. If asystole recurs frequently, ventricular pacing and atropine (and similar compounds) may be helpful for performing RF ablation procedure.

## Consent

Written informed consent was obtained from the patient for publication of this case report and accompanying images. A copy of the written consent is available for review by the Editor-in-Chief of this journal.

## Abbreviations

AVNRT: Atrioventricular nodal reentrant tachycardia; AVRT: Atrioventricular reentrant tachycardia; CS: Coronary sinus; EPS: Electrophysiologic study; F: Failure; GP: Ganglionated plexus; LSAGP: Left superior atrial ganglionated plexus; LSPV: Left superior pulmonary; LVFW: Left ventricular free wall; RF: Adiofrequency; N: No; PAF: Paroxysmal atrial fibrillation; PMLAGP: Posteromedial left atrial ganglionated plexus; PLLAGP: Posterolateral left atrial ganglionated plexus; S: Success; SP: Slow pathway; SVT: Supraventricular tachycardia; VA: Ventriculoatrial; Y: Yes.

## Competing interests

The authors declare that they have no competing interests.

## Authors’ contributions

SHY, MYC analyzed and interpreted the patient’s clinical and laboratory data. HSH, MX, XJW performed the literature review and wrote the manuscript. RX critically revised the manuscript. All authors have read and approved the final version of this manuscript.
